# Hippocampal Metabolite Profiles in Two Rat Models of Autism: NMR-Based Metabolomics Studies

**DOI:** 10.1007/s12035-020-01935-0

**Published:** 2020-05-28

**Authors:** B. Toczylowska, E. Zieminska, P. Senator, J. W. Lazarewicz

**Affiliations:** 1 Nalecz Institute of Biocybernetics and Biomedical Engineering, Polish Academy of Sciences, Warsaw, Poland; 2 Mossakowski Medical Research Centre, Polish Academy of Sciences, A. Pawinskiego st, 02-106 Warsaw, Poland

**Keywords:** Autism, Metabolomics, NMR spectroscopy

## Abstract

Autism spectrum disorders (ASDs) are increasingly being diagnosed. Hypotheses link ASD to genetic, epigenetic, or environmental factors. The role of oxidative stress and the imbalance between excitatory and inhibitory neurotransmission in the pathogenesis of ASD has been suggested. Rats in which ASD symptoms are induced by valproate (VPA) or thalidomide (THAL) application in utero are useful models in ASD studies. Our study investigated whether rats in ASD models show changes in metabolite levels in the brain consistent with the hypothetical pathomechanisms of ASD. Female rats were fed one dose of 800 mg/kg VPA or 500 mg/kg THAL orally on the 11th day of gestation, and 1-month offspring were used for the experiments. Metabolic profiles from proton nuclear magnetic resonance spectroscopy of hydrophilic and hydrophobic extracts of rat hippocampi were subjected to OPLS-DA statistical analysis. Large differences between both models in the content of several metabolites in the rat hippocampus were noticed. The following metabolic pathways were identified as being disturbed in both ASD models: steroid hormone biosynthesis; fatty acid biosynthesis; the synthesis and degradation of ketone bodies; glycerophospholipid metabolism; cholesterol metabolism; purine metabolism; arginine and proline metabolism; valine, leucine, and isoleucine biosynthesis and degradation. These results indicate disorders of energy metabolism, altered structure of cell membranes, changes in neurotransmission, and the induction of oxidative stress in the hippocampus. Our data, consistent with hypotheses of ASD pathomechanisms, may be useful in future ASD studies, especially for the interpretation of the results of metabolomics analysis of body fluids in rat ASD models.

## Introduction

Autism spectrum disorder (ASD) is an increasingly emerging disease that appears worldwide, and the prevalence of ASD ranges from 25 to 110 cases per 10,000 children, depending on the country [1]. It seems that ASD occurs 2–3 times more often in boys than that in girls [2]. The etiology of autism disease is complex and not yet fully explained. There are hypotheses linking symptoms of ASD with genetic [3–5], epigenetic, or environmental factors [1]. Epigenetic factors could modify the expression of mRNA or miRNA, which could affect the conformation and content of proteins involved in body functions [6]. Environmental factors, due to their toxicity, could affect not only young subjects but also fetuses through toxic influences on pregnant mothers [7–9]. Drugs [1], as well as disturbances in the levels of metal ions (i.e., zinc) [10, 11], in hormonal systems [12] and in brain amino acid–mediated neurotransmission [13] could also be implicated in ASD etiology and/or pathogenesis. According to this interpretation, behavioral disorders observed in ASD may result from changes at the level of gene expression and in the level and activity of specific proteins, which may consequently be reflected in changes in the level of certain metabolites in the brain.

There are several analytical methods that allow us to study the abovementioned factors. One of them is nuclear magnetic resonance (NMR). Magnetic resonance spectroscopy (MRS) can be applied in vivo to examine the content of various metabolites in the brains of autistic children, while NMR can be used ex vivo to study biofluids such as serum, urine, or saliva [14, 15]. MRS is a low-resolution study and does not allow the recognition of all amino acids and lipids. One of the most complex methods for the study of small molecules is metabolomics based on NMR spectroscopy. A critical advantage of NMR spectroscopy is its ability to detect many compounds present in the examined sample in a single experiment. Additionally, this study method makes it possible to perform quantitative analysis with the use of a single reference compound. NMR spectroscopy is most useful if no single biomarker could be identified for differential diagnosis. It could also be applied to study concentrations of neuroactive amino acids, such as glutamate, GABA, and glutamine, as well as taurine, which, according to the glutamatergic hypothesis of ASD, could be candidates for biomarkers [13, 16]. Previous pioneering studies of autism and schizophrenia using NMR spectroscopy–based metabolomics led to the disclosure of disturbances in glutamate and taurine concentrations in the urine of children with ASD [16–18]. However, detailed tissue analysis, in particular of ex vivo brain extracts using NMR spectroscopy in practice, is limited to studies using animal models of various diseases.

There are numerous animal models of autism used in laboratory studies, including genetically modified animals [19] or those based on the use of specific substances, e.g., teratogenic drugs valproic acid (VPA) or thalidomide (THAL). Drugs in these animal models of ASD are administered to mothers during the critical period of gestation [20, 21]. Similar neurodevelopmental effects in offspring can be achieved by causing thyroid hormone [22] or zinc [23] deficiency in pregnant mothers, as well as by inducing specific inflammation [24, 25]. In all these models, offspring exhibit behavior similar to ASD, validated using animal behavioral tests [26–29]. The advantage of these models compared with transgenic animals is the similarity of behavioral disorders induced in this way to the idiopathic symptoms of autism [20, 21]. It seems that these animal models of autism could be useful for the study of the content of biochemical compounds in the brain to test the compliance of observed changes with hypotheses regarding the pathogenesis of ASD. Such studies are impossible to carry out on humans.

In our previous study [30], in which NMR spectroscopy was used in addition to HPLC, we showed changes in glutamate, glutamine, and GABA levels in the rat hippocampus in VPA- and THAL-induced models of autism. However, the results obtained differed depending on the analytical method used, the experimental group, and the animal sex. This made it difficult to draw useful conclusions for the determination of the role of changes in excitatory and inhibitory neurotransmission in the pathogenesis of autistic-like behaviors in the animals studied. Therefore, based on this previous experience, we decided to focus our further study on only NMR spectroscopy and to try to identify metabolites other than amino acid neurotransmitters, both hydrophilic and hydrophobic, that can be determined using this method, the content of which in the hippocampus is changed in rat ASD models*.*

The aim of the current study was to better characterize metabolic changes in the rat brain in chemically induced ASD models, which would be useful for the validation of these models for further studies on the pathogenesis of ASD. We intended to check if by using these models we could detect changes in substance concentrations that could indicate the contribution of postulated mechanisms associated with the pathogenesis of autism, such as oxidative stress or an imbalance between excitatory and inhibitory neurotransmission. Another goal was to attempt to identify metabolites whose levels undergo similar changes in two selected experimental models. In the future, this may help in the identification of potential ASD biomarkers. In this study, we used two established rat ASD models prenatally induced by the application of VPA and THAL. NMR spectrometry was used to determine the ex vivo content of a number of detectable hydrophilic and hydrophobic metabolites in hippocampal extracts of juvenile 1-month-old rats.

## Methods

### Animal Models of Autism

Experiments were performed using Wistar rats of both sexes. The animals were bred in the Animal Colony of the Mossakowski Medical Research Centre, Polish Academy of Sciences, in Warsaw. The animals were provided water and fed ad libitum and kept on a 12-h dark/light cycle at room temperature with a constant humidity of approximately 60%. All procedures involving animals were in accordance with the EC Directive for the use of experimental animals 2010/63/EU from 2010, with further modifications, and the national law.

They were approved by the Fourth Local Ethical Committee in Warsaw (resolution no. 43/2015 of May 22, 2015).

In this project, two chemical teratogenic models of autism were used. The procedure was performed exactly as previously described [30]. Female rats on the 11th day of gestation were fed one dose of 800 mg/kg b.w. VPA or 500 mg/kg b.w. THAL. VPA was mixed with 1 ml of saline, THAL was mixed with vegetable oil, and both were administered orally via an intragastric tube. Control animals were fed 1 ml of a mixture of oil and saline, 1:1 v/v [20, 21]. The 31 (± 2)-day-old Wistar rats of both sexes (F: female and M: male) were used for NMR experiments. The use and distribution in groups of all animals are shown in Table 1. The rats of each group came from two litters. Each group had a different number of female and male rats. There were also animals used in other studies not described in this manuscript. The total number of pups in the two litter of each group was as follows: in the control group, 27 (16F:11M); in the VPA group, 22 (11F:11M); and in THAL group, 19 (7F:12M). Finally, in these studies, there were 11 (6F + 5M) control animals, 11 (5F + 6M) VPA-treated animals, and 13 (4F+9M) THAL-treated animals.

**Table 1:** The use and distribution in groups of all animals from the two litters used in this study

		Control			VPA			THAL		
		*∑*	M	F	*∑*	M	F	*∑*	M	F
I litter	Total animals	13	4	9	13	6	7	12	6	6
	This study	4	3	1	7	3	4	6	3	3
	Other study	9	1	8	6	3	3	6	3	3
II litter	Total animals	14	7	7	9	5	4	7	6	1
	This study	7	5	2	4	3	1	7	6	1
	Other study	7	2	5	5	2	3	0	0	0

*VPA* valproate treated group, *THAL* thalidomide treated group, *M* male, *F* female animals

### Sample Preparation

After decapitation, one hippocampus was removed and homogenized for 2 min by hand in 500 μl of ice-cold saline using a plastic/Teflon homogenizer. Then, 400-μl aliquots of fresh hippocampal homogenates were extracted for NMR studies using the Bligh and Dyer method [31], with slight modification, exactly according to the procedure described previously [30]. Briefly, the homogenates were vortexed for 1 min with 1875 μl of a mixture of 99% methanol, 98% chloroform, and 36% HCl, 40:20:1 (v/v). As the next step, 625 μl chloroform was added, and the mixture was again vortexed for 1 min. After that, 625 μl of water was added and vortexed for 1 min. Then, the mixture was centrifuged at 2000×*g* for 15 min using a swing-out rotor to obtain three phases: upper, water/methanol containing substances diluted in water; lower, containing lipids; and middle, containing proteins. Upper and lower phases were extracted for the NMR examination. Middle phases were collected to assess total protein content in samples (using the Lowry test) to normalize the concentration of compounds obtained in the hippocampus. The water/methanol phase of the sample was dried using nitrogen. Dry residues were then diluted in 700 μl of D_2_O and immediately tested. Additionally, the lipid phase was dried using nitrogen, dissolved in 700 μl of CDCl_3_, and immediately tested.

### Spectra Acquisition

The pH of the samples was adjusted to 7.5 ± 0.2 using HCl. 3-Trimethylsilyl propionic acid (TSP) at a final concentration of 1 mM was used as an internal reference for the normalization of all spectra and quantitative statistical analysis. All NMR spectra of hydrophilic compounds were acquired at 25 °C using an Avance III HD 500 MHz (Bruker, Germany) spectrometer. Excitation sculpting [32] was used to suppress the water signal while minimizing phase distortion of the spectrum and utilized a 2-ms square inversion pulse in a double pulse field gradient spin echo. Line broadening of 0.5 and baseline and phase corrections were applied to each spectrum using software implemented in the spectrometer. Hydrophobic compounds were measured using a single pulse sequence at 20 °C and 128 transients with a 5-s repetition time.

All spectra were first both baseline and phase corrected and analyzed. There were 98 signals of hydrophilic and 45 hydrophobic functional groups of compounds. Signal assignments were performed using our own database of spectra of reference compounds and literature data, considering correction for the modified extraction method [33]. For the confirmation of signal assignment, other NMR experiments were performed as follows: 1H-1H COSY, 31P, 1H-31P HSQC. For further statistical analyses, we selected the 55 hydrophilic and 24 hydrophobic most isolated NMR signals that represent all assigned and unassigned compounds, and their magnitudes were measured and normalized to the TSP or CDCl_3_ rest signal prior to statistical analyses.

### Statistical Analysis

Univariate statistical analyses, one-way ANOVA, and two-way ANOVA tests followed by Dunn’s corrections were performed using the SigmaPlot 12.5 software package (Systat Software, Inc.). Two-way ANOVA was carried out for group and sex factors. A *p* value lower than 0.05 was considered significant. Statistical multivariate analyses (MVAs) of principal component analysis (PCA) and orthogonal partial least squares discriminant analysis (OPLS-DA) were described in detail in our previous publication [30]. In the analysis, the X-matrix (independent variables) represents all data obtained from NMR spectral analysis, and the Y-matrix (dependent variable) represents all groups [34]. Models were validated using an analysis of variance of cross-validation estimation (CV-ANOVA). The variable importance in the projection (VIP) value of each variable in the model was calculated. MVA was performed using the SIMCA software package, ver. 15, Sartorius Stedim Data Analytics AB, Sweden [35].

## Results

The content of metabolites in the samples was expressed as the magnitude of their NMR signals, which is known to correspond to the concentration of the compound, normalized to the protein concentration in the sample. Fifty-five hydrophilic NMR signals representing various compounds were statistically analyzed, of which ten signals were not assigned to the compounds. In turn, 24 hydrophobic NMR signals representing various compounds or compound complexes were statistically analyzed, of which three signals were not assigned to the compounds. PCA was used to identify outliers. Two outliers were identified: one sample in the VPA group and one sample in the THAL group, and their data were removed from further analyses. Finally, in statistical analyses, the number of samples in each group was as follows: control, 11; VPA, 10; and THAL, 12. Because it is difficult to identify a single biomarker that, with high sensitivity and specificity, distinguishes a patient from the healthy population, the entire dataset of compounds was subjected to multivariate OPLS-DA analysis. When added to the analysis of the group parameter (Y-matrix), we did not observe any influence of the sex parameter on the metabolic profile MVA. The result of the MVA was the same, regardless of whether we considered sex and the group or just the group.

### Hydrophilic Compounds

Considering hydrophilic substances, statistically significant differences between the VPA-treated and control groups (Table 2) were observed for hypoxanthine, 3-OH-butyrate, and glutathione. The concentrations of 3-OH-butyrate and glutathione decreased in the VPA group, while the concentration of hypoxanthine increased. Statistically significant differences in the concentrations of hydrophilic compounds between the control and THAL groups (Table 1) were observed for 8-hydroxyadenine, hypoxanthine, GMP, guanine, and xanthine complex signals; thymine, allantoin, myo-inositol, taurine, taurine, and phosphoethanolamine complex signals; glycerophosphorylcholine, phosphorylcholine, choline, creatinine, L-cysteic acid, creatine, aspartate, L-glutamine, L-glutamate, acetate, NAA, GABA, L-alanine, lactate, L-threonine, and L-valine; overlapping signals from leucine and isoleucine; and overlapping signals from 3-OH-valerate and methylmalonate, and unassigned signals Nos. 2 and 4. All signal magnitudes except guanine, xanthine, and overlapping signals from 3-OH-valerate and methylmalonate were higher in the THAL group than those in the control group.

**Table 2:** Hydrophilic compounds found in NMR analysis of hippocampus extracts

Chemical shift (ppm)	Compound	VPA			THAL		
		% of control	ANOVA	VIP value	% of control	ANOVA	VIP value
9.29	Unassigned 1	93	*F*(1,20) = 0.0504	1.25	133	*H*(1) = 0.750	1.0
						*p* = 0.387	
			*p* = 0.825				
9.24	Unassigned 2	84	*H*(1) = 2.623		124	*F*(1,22) = 4.369	
			*p* = 0.105			*p* = 0.049*	
8.96	(–C(4)H–) N-Methylnicotinamide	104	*F*(1,20) = 0.0735		122	*H*(1) = 2.182	
						*p* = 0.140	
			*p* = 0.789				
8.70	(=C(2)H–) 8-Hydroxyadenine	94	*H*(1) = 0.0198	1.32	160	*F*(1,22) = 4.571	1.44
			*p* = 0.888			*p* = 0.044*	
8.68	(–C(2)H–) Inosine	105	*F*(1,20) = 0.0827		110	*F*(1,22) = 0.195	
						*p* = 0.663	
			*p* = 0.777				
8.59	(–C(8)H–) IMP	84	*F*(1,20) = 0.824	1.08	119	*F*(1,22) = 0.708	
			*p* = 0.375			*p* = 0.410	
8.45	(–C(8)H–) Hypoxanthine	141	*H*(1) = 4.170	1.84	243	*F*(1,22) = 14.808	1.55
			*p* = 0.041*				
						*p* < 0.001*	
8.29	(–C(8)H–) GMP	72	*H*(1) = 0.838	1.04	155	*H*(1) = 4.125	
			*p* = 0.360			*p* = 0.042*	
8.24	Unassigned 3	55	*H*(1) = 3.354	1.39	87	*H*(1) = 0.136	
			*p* = 0.067			*p* = 0.712	
7.96	(–C(4)H–) Cytosine	102	*F*(1,20) = 0.0148		118	*F*(1,22) = 1.067	
						*p* = 0.313	
			*p* = 0.904				
7.67	(–C(8)H–) Guanine/(–C(2)H–) xanthine	107	*H*(1) = 0.317		36	*H*(1) = 9.470	1.69
			*p* = 0.573			*p* = 0.002*	
7.43	Unassigned 4	84	*H*(1) = 1.607		131	*H*(1) = 4.640	
			*p* = 0.205			*p* = 0.031*	
7.33	(–C(6)H–) Thymine	94	*H*(1) = 0.124		144	*F*(1,22) = 10.350	
			*p* = 0.725				
						*p* = 0.004*	
7.10	Unassigned 5	83	*H*(1) = 1.790		108	*F*(1,22) = 0.397	
			*p* = 0.181			*p* = 0.536	
6.92	Unassigned 6	86	*H*(1) = 0.972		116	*F*(1,22) = 0.690	
			*p* = 0.324			*p* = 0.415	
6.85	(–CH=CH–) Fumarate	96	*F*(1,20) = 0.0547		125	*F*(1,22) = 1.858	
						*p* = 0.187	
			*p* = 0.818				
6.80	(–C(2,6)H–) Tyrosine	87	*H*(1) = 0.496		112	*H*(1) = 0.640	
						*p* = 0.424	
			*p* = 0.481				
6.22	(–C(1′)H–) AMP	90	*F*(1,20) = 0.429		130	*F*(1,22) = 2.551	
			*p* = 0.520			*p* = 0.125	
5.98	Unassigned 7	103	*F*(1,20) = 0.0721		113	*F*(1,22) = 0.933	
						*p* = 0.345	
			*p* = 0.791				
5.39	(–C(4)H–) Allantoin	60	*H*(1) = 0.526		340	*H*(1) = 7.542	1.56
			*p* = 0.468			*p* = 0.006*	
4.19	(–C(1)H_2_–) Phosphorylcholine	90	*H*(1) = 0.0198		142	*H*(1) = 3.186	1.10
			*p* = 0.888			*p* = 0.074	
4.04	(–C(2)H_2_–) Glycolic acid	67	*H*(1) = 0.124	1.21	117	*F*(1,22) = 0.249	
			*p* = 0.725			*p* = 0.623	
3.86	Unassigned 8	92	*H*(1) = 0.972		110	*F*(1,22) = 0.527	
			*p* = 0.324			*p* = 0.477	
3.79	(–C(1)H_2_–) Guanidinoacetate	102	*H*(1) = 0.317		144	*H*(1) = 1.515	
			*p* = 0.573			*p* = 0.218	
3.71	Unassigned 9	136	*F*(1,20) = 1.775	1.72	754	*H*(1) = 3.409	1.20
			*p* = 0.198			*p* = 0.065	
3.66	Unassigned 10	80	*F*(1,20) = 2.513	1.21	94	*F*(1,22) = 0.224	
			*p* = 0.129			*p* = 0.641	
3.55	(–C(2)H_2_–) Glycine	91	*H*(1) = 1.790	1.36	133	*F*(1,21) = 3.384	
			*p* = 0.181			*p* = 0.081	
3.52	(–C(6,4)H–) Myo-inositol	110	*F*(1,20) = 0.467		148	*F*(1,22) = 11.197	1.07
			*p* = 0.503				
						*p* = 0.003*	
3.42	(–C(3)H_2_–) Taurine	85	*H*(1) = 0.972		141	*H*(1) = 6.367	1.06
			*p* = 0.324			*p* = 0.012*	
3.35	((–CH–)_6_) Scyllo-inositol	87	*H*(1) = 1.433		65	*F*(1,22) = 2.020	1.06
			*p* = 0.231			*p* = 0.170	
3.25	(–C(3)H_2_–) Taurine/(–C(2)H_2_–) Phosphoethanolamine	90	*H*(1) = 179		163	*H*(1) = 8.015	1.28
			*p* = 0.673			*p* = 0.005*	
3.23	(–N–(CH_3_)_3_) Phosphorylcholine/glycerophosphorylcholine	90	*F*(1,20) = 0.562		177	*F*(1,22) = 21.144	1.33
			*p* = 0.463				
						*p* < 0.001*	
3.20	(–N–(CH_3_)_3_) Choline	72	*H*(1) = 3.352		196	*F*(1,22) = 13.111	1.17
			*p* = 0.067				
						*p* = 0.002*	
3.14	(–N–(CH_3_)_3_) Creatinine	117	*F*(1,20) = 0.596	1.21	212	*F*(1,22) = 16.402	1.51
			*p* = 0.450				
						*p* < 0.001*	
3.09	(–C(2)H–) L-cysteic acid	94	*F*(1,20) = 0.205		167	*F*(1,22) = 14.240	1.17
			*p* = 0.656				
						*p* = 0.001*	
3.07	(–C(6)H_3_–) Creatine	87	*F*(1,20) = 0.774		141	*F*(1,22) = 5.349	1.03
			*p* = 0.390			*p* = 0.031*	
2.97	(–C(3)H_2_–) Aspartate	95	*H*(1) = 0.0		145	*F*(1,22) = 9.080	1.09
			*p* = 1.000			*p* = 0.007*	
2.88	(N–(CH_3_)_2_) Dimethylamine	93	*H*(1) = 0.0198		124	*F*(1,22) = 2.563	
			*p* = 0.888			*p* = 0.124	
2.68	(–C(2)–CH_3_) Pyruvate/(–C(2,3)H_2_–) succinate	93	*F*(1,20) = 0.329		115	*F*(1,22) = 1.244	
			*p* = 0.573			*p* = 0.277	
2.63	(–C(4)H_2_–) L-Glutamate	113	*F*(1,20) = 0.609		154	*F*(1,22) = 8.600	
			*p* = 0.445			*p* = 0.008*	
2.49	(–C(4)H_2_–) L-Glutamine	88	*F*(1,20) = 1.129		138	*F*(1,22) = 10.721	
			*p* = 0.301				
						*p* = 0.004*	
2.38	(–C(10)H_2_–) Glutathione	74	*H*(1) = 7.934	1.34	91	*F*(1,22) = 1.624	
						*p* = 0.217	
			*p* = 0.005*				
2.22	(–C(15)H_3_) NAAG	81	*H*(1) = 0.496		21	*H*(1) = 2.562	1.69
			*p* = 0.481			*p* = 0.109	
2.10	(–C(2)H_3_) Acetate	92	*F*(1,20) = 0.313		168	*F*(1,22) = 15.804	1.27
			*p* = 0.582				
						*p* < 0.001*	
2.04	(–C(7)H_3_) NAA	101	*F*(1,20) = 0.0741		157	*F*(1,22) = 12.394	1.18
			*p* = 0.932			*p* < 0.002*	
1.96	(–C(3)H_2_–) GABA	94	*H*(1) = 0.124		155	*F*(1,22) = 13.302	1.12
			*p* = 0.725				
						*p* = 0.002*	
1.78	(–C(5)H_2_–) Lysine	91	*H*(1) = 0.0446		108	*H*(1) = 0.545	
			*p* = 0.833			*p* = 0.460	
1.55	(–C(3)H_3_) L-Alanine	97	*F*(1,20) = 0.044		132	*F*(1,22) = 5.281	
			*p* = 0.836			*p* = 0.032*	
1.42	(–C(3)H_3_) Lactate	76	*F*(1,20) = 2.150	1.07	149	*H*(1) = 5.186	1.02
			*p* = 0.159			*p* = 0.023*	
1.35	(–C(3)H_3_) L-Threonine	88	*F*(1,20) = 0.818		145	*F*(1,22) = 7.909	
			*p* = 0.377			*p* = 0.010*	
1.20	(C(3)–(CH_3_)_2_) 3-hydroxyisovalerate/(–C(2)–CH_3_) methylmalonate	99	*H*(1) = 0		0	*H*(1) = 6.540	1.11
			*p* = 1.000			*p* = 0.011*	
1.18	(–C(2)H_2_–) 3-Hydroxybutyrate	41	*H*(1) = 4.765	2.50	334	*H*(1) = 2.004	
			*p* = 0.029*			*p* = 0.157	
1.14	(–C(3)–(CH_3_)_2_) 2- oxoisovalerate	50	*H*(1) = 3.615	2.05	308	*H*(1) = 2.761	
			*p* = 0.057			*p* = 0.097	
1.08	(–C(4)H_3_) L-Valine	98	*H*(1) = 0.00496		144	*H*(1) = 6.367	1.05
			*p* = 0.944			*p* = 0.012*	
0.96	(–C(5.6)H_3_) L-Leucine/(–C(3)–CH_3_) isoleucine	102	*H*(1) = 0.124		148	*F*(1,22) = 7.383	1.12
			*p* = 0.725			*p* = 0.013*	

Only VIP values > 1 obtained in MVA analysis are presented

^*^Statistically significant

**Fig. 1:**
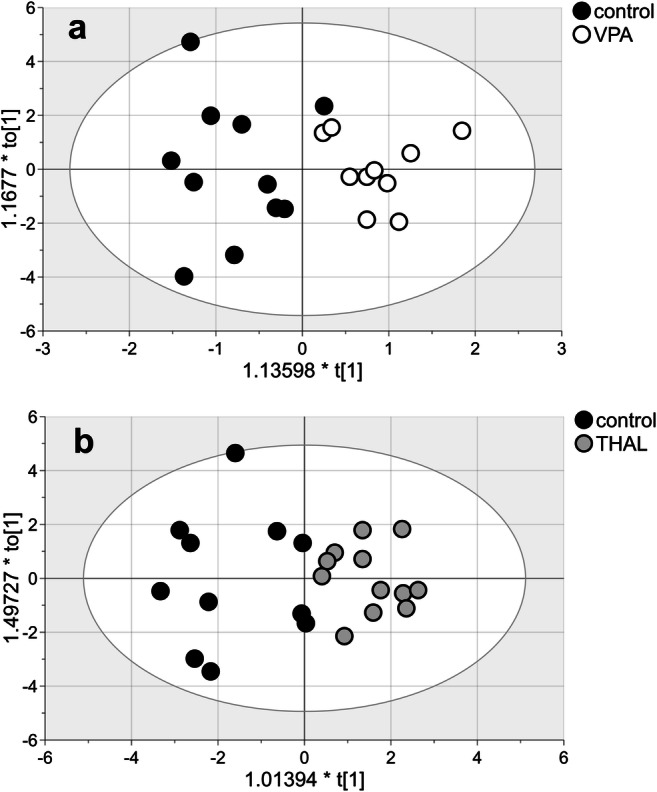
The score plot of the two-component OPLS-DA model for hydrophilic compounds of NMR data for VPA vs control (**a**) and THAL vs control group (**b**); to[1] represents within-class variation in the first orthogonal component, whereas t[1] represents between-class variation in the first predictive component. Ellipse represents Hotelling’s T2 with 95% confidence in score plots

MVA of the hydrophilic compounds detected in the hippocampal homogenates collected from the VPA-treated group allowed us to build a valid model (*p* = 0.036) (Fig. 1a). The model consisted of two components: one predictive and one orthogonal to the data. *R*^2^ for this model was 0.733, and *Q*^2^ was 0.455. The model fits the data well and was good for prediction. Samples were classified correctly in 95.2% of their groups (*p* < 0.001): 90.91% in the control group (10 out of 11) and 100% in the VPA group. MVA indicated significant group differentiation (VIP > 1) of the following compounds: 3-hydroxybutyrate, 2-oxoisovalerate, lactate, glutathione, creatinine, glycine, glycolic acid, GMP, hypoxanthine, IMP, 8-hydroxyadenine, and unassigned compound signals Nos. 1, 3, 9, and 10 (Table 2).

MVA of the hydrophilic compounds for the THAL group also allowed us to build a valid model (*p* = 0.007). The model consisted of two components: one predictive and one orthogonal to the data (Fig. 1b). *R*^2^ for this model was 0.721, and *Q*^2^ was 0.527. Samples were classified correctly to their groups in 87% (*p* < 0.001): 72.7% (8 out of 11) in the control group and 100% in the THAL group. The following substances were found: L-valine, leucine/isoleucine, GABA, NAA, acetate, lactate, 3-hydroxyisovalerate and methylmalonate, NAAG, aspartate, choline, creatine, L-cysteic acid, creatinine, taurine, myo-inositol, allantoin, hypoxanthine, taurine/phosphoethanolamine, scyllo-inositol, phosphocholine/glycerophosphocholine, 8-hydroxyadenine, phosphorylcholine, guanine and xanthine overlapped signal, and unassigned compound signals Nos. 1 and 9 (Table 2).

### Hydrophobic Compounds

The analysis of the content of hydrophobic substances showed statistically significant differences between the VPA group and the control group (Table 3) in the common signal of the saturated/monounsaturated/polyunsaturated fatty acids (FAs/MUFAs/PUFAs) complex, estriol, and 24-hydroxycholesterol. The concentrations of all these substances decreased in the VPA group compared with those in the control group.

**Table 3:** Hydrophobic compounds found in NMR analysis of hippocampus extracts

Chemical shift (ppm)	Compound/functional group	VPA			THAL		
		% of control	ANOVA	VIP value	% of control	ANOVA	VIP value
6.87	Unassigned 1	85	*F*(1,21) = 2.920	1.06	86	*F*(1,23) = 1.665	
			*p* = 0.103			*p* = 0.210	
6.09	(–HC(2,4)=) Estriol	78	*F*(1,21) = 5.222	1.35	71	*F*(1,23) = 9.735	1.37
			*p* = 0.033*			*p* = 0.005*	
5.87	(–C(4)H=C(5)H–) Testosterone	165	*H*(1) = 0.683	2.27	53	*H*(1) = 1.137	1.52
			*p* = 0.407			*p* = 0.286	
5.14	(–HC=CH–) Olefinic group in MUFA	93	*F*(1,21) = 0.228		96	*H*(1) = 4.720	1.35
			*p* = 0.638			*p* = 0.030*	
5.03	Unassigned 2	82	*F*(1,21) = 0.690	1.67	117	*F*(1,23) = 0.582	1.16
			*p* = 0.416			*p* = 0.454	
4.70	Unassigned 3	68	*H*(1) = 2.804	1.54	112	*H*(1) = 0.0680	
						*p* = 0.794	
			*p* = 0.094				
4.15	(–C(2)H–) Sphingomyelin (d18:1/16:0)	103	*F*(1,21) = 0.0719		119	*F*(1,23) = 2.359	
						*p* = 0.139	
			*p* = 0.791				
3.88	(–CH_2_–CH_2_–N(–CH_3_)_3_) L-α-Phosphatidylcholine (16:0/18:2)	104	*F*(1,21) = 0.145		124	*H*(1) = 3.123	
			*p* = 0.708			*p* = 0.077	
3.68	(ROCH_2_–CHOH–CH_2_OH) in glyceryl group in 1-MG	112	*F*(1,21) = 0.556		145	*F*(1,23) = 7.328	1.10
			*p* = 0.465			*p* = 0.013*	
3.24	(–O–CH_2_–CH_2_–NH_3_) L-α-Phosphatidylethanolamine (18:0/18:0)	91	*F*(1,21) = 0.515		101	*F*(1,23) = 0.00762	
			*p* = 0.483				
						*p* = 0.932	
2.30	(–OCO–CH_2_–), (–COOH–CH_2_–) Acyl groups in 1,3-DG, 1-MG, and FA	107	*F*(1,21) = 0.262		93	*F*(1,23) = 0.225	
			*p* = 0.614			*p* = 0.643	
2.22	(–C(α)H_2_–) in saturated FA, MUFA, and PUFA	64	*F*(1,21) = 11.580	1.60	61	*F*(1,23) = 10.494	1.37
						*p* = 0.005*	
			*p* = 0.003*				
2.00	(–CH_2_–CH=CH–) in acyl groups of FA and phospholipids	88	*F*(1,21) = 1.093		103	*F*(1,23) = 0.0717	
			*p* = 0.310			*p* = 0.792	
1.70	(–C(15)H_2_–) Palmitic acid in L-α- lysophosphatidylcholine and FA	84	*H*(1) = 2.381	1.17	140	*H*(1) = 3.545	1.07
			*p* = 0.123			*p* = 0.060	
1.60	(–OCO–CH_2_–CH_2_–) in acyl groups in 1,3-DG, 1-MG, and FA	89	*F*(1,21) = 1.516		106	*F*(1,23) = 0.359	
			*p* = 0.233			*p* = 0.555	
1.28	(−(CH_2_)_n_–) in acyl groups in FA	95	*F*(1,21) = 0.365		106	*F*(1,23) = 0.509	
			*p* = 0.552			*p* = 0.483	
1.08	(–C(21)H_3_) Cholestenol	101	*F*(1,21) = 0.0148		124	*p* = 0.029*	
			*p* = 0.904				
0.98	(–C(ω)H_3_) in PUFA/MUFA	108	*F*(1,21) = 0.385		131	*F*(1,23) = 6.927	1.21
			*p* = 0.542			*p* = 0.015*	
0.93	(–C(21)H_3_) Free cholesterol and 25-hydroxycholesterol	104	*F*(1,21) = 0.118		125	*F*(1,23) = 6.076	1.10
			*p* = 0.734			*p* = 0.022*	
0.88	(–CH_3_) in acyl groups of FA and sterols	93	*F*(1,21) = 1.000		107	*F*(1,23) = 0.805	
			*p* = 0.329			*p* = 0.379	
0.72	(–C(18)H_3_) 24-Hydroxycholesterol	70	*H*(1) = 7.788	1.34	83	*F*(1,23) = 2.228	
			*p* = 0.005*			*p* = 0.150	
0.69	(–C(18)H_3_) Free cholesterol and cholesterol esters	103	*F*(1,21) = 0.0971		121	*F*(1,23) = 5.099	1.00
						*p* = 0.034*	
			*p* = 0.759				
0.62	(–C(18)H_3_) Progesterone	87	*F*(1,21) = 0.233	1.03	113	*F*(1,23) = 0.240	1.28
			*p* = 0.635			*p* = 0.629	
0.53	(–C(18)H_3_) Lathosterol	108	*F*(1,21) = 0.950		115	*F*(1,23) = 2.759	
			*p* = 0.341			*p* = 0.111	

Only VIP values > 1 obtained in MVA analysis are presented

^*^Statistically significant

Statistically significant differences between the control and the THAL group (Table 3) were observed for estriol, the olefinic group in MUFAs, the FAs/MUFAs/PUFAs complex signal, cholestenol, free cholesterol and 25-hydroxycholesterol, free cholesterol and cholesterol esters, and the glyceryl group in 1-MG and 1,2-DG. All compound concentrations increased in the THAL group compared with those in the control group except for the FAs/MUFAs/PUFAs complex signal and estriol, whose concentrations decreased.

**Fig. 2:**
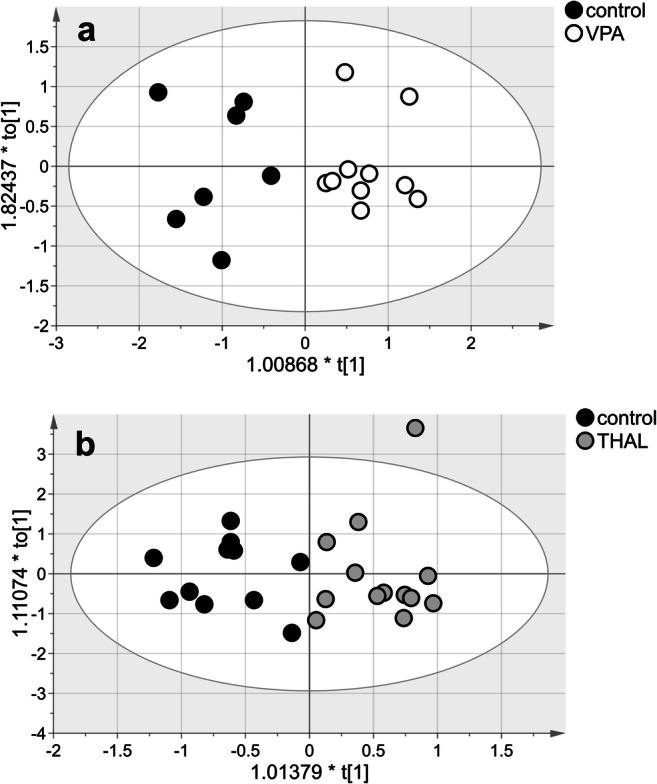
The score plot of the two-component OPLS-DA model for hydrophobic compounds of NMR data for VPA vs control (**a**) and THAL vs control group (**b**); to[1] represents within-class variation in the first orthogonal component, whereas t[1] represents between-class variation in the first predictive component. Ellipse represents Hotelling’s T2 with 95% confidence in score plots

MVA of the lipid data for the VPA group allowed us to build a valid model (*p* = 0.04). The model consisted of two components: one predictive and one orthogonal to the data (Fig. 2a). *R*^2^ for this model was 0.833, and *Q*^2^ was 0.546. The model fits the data well and was good for prediction. Samples were classified correctly (*p* < 0.001) in 100% of their groups. MVA indicated significant group differentiation (VIP > 1) of the following substances: estriol, testosterone, progesterone, saturated FAs/MUFAs/PUFAs complex signal, palmitic acid in LPtdC and FAs, 24-hydroxycholesterol, and unassigned compound signals Nos. 1–3. Among substances with altered concentrations in the VPA-treated group, only testosterone concentration was higher, while concentrations of all other compounds were lower compared with those in the control group (Table 3).

MVA of the lipid data for the THAL group also allowed us to build a valid model (*p* = 0.04). The model consisted of three components: one predictive and two orthogonal to the data (Fig. 2b). *R*^2^ for this model was 0.778, and *Q*^2^ was 0.518. Samples were classified correctly (*p* < 0.001) in 100% of their groups. MVA analysis indicated significant group differentiation (VIP > 1) signals from estriol, testosterone, progesterone, MUFAs, 1-MG, palmitic acid in LPtdC and FAs/MUFAs/PUFAs complex signal, free cholesterol/cholesterol ester complex signal, free cholesterol/25-hydroxycholesterol complex signal, and unassigned signal No. 2 (Table 2). Among substances with changed concentrations in the THAL-treated group, only testosterone, estriol, and olefinic acid in MUFAs and FAs/MUFAs/PUFAs concentrations were decreased, while concentrations of all other compounds were increased compared with those in the control group (Table 3).

## Discussion

In our studies, instead of genetic models, only pharmacological models of ASD on rats were used. This is due to our priority interest in the role of environmental factors, especially neurotoxic ones, in the etiopathogenesis of autism. Both VPA and THAL are used in therapy, so it is important to better understand their adverse effects on the fetus in the early stages of pregnancy, which may result in ASD in the offspring. In addition, we expected that a comparison of metabolomic changes in both pharmacological models could help clarify whether both models can be used alternatively, e.g., for testing new therapies. In this work, research focused on the hippocampus, one of the brain regions whose developmental disorders are particularly strongly associated with the pathogenesis of behavioral disorders in ASD [36]. This is a continuation and extension of the scope of our previous studies [30], in which only changes in the concentration of neuroactive amino acids in the hippocampus in rat ASD models were analyzed.

Our discussion of the results obtained in this study mainly focuses on the role of the substances identified in the NMR spectra whose content in rat hippocampus homogenates from experimental groups proved to be significantly (VIP > 1) different from that in the control group in the MVA and whose changes can be related to hypothetical mechanisms implicated in the pathogenesis of ASD. When necessary, we also discuss the results for some other substances.

### Hydrophobic and Hydrophilic Compounds in Rat ASD Models and Their Potential Role in Excitation/Inhibition Imbalance in the Hippocampus

In both experimental groups, MVA showed a statistically significant decrease in the levels of FAs/MUFAs/PUFAs. Fatty acids (FAs), carboxylic acids with aliphatic chains, are the main components of lipids that form the cell membrane and thus play an important role in the structure and function of the nervous system. FA composition affects the activity of ion channels and receptors. High levels of n-6 PUFAs are generally pro-inflammatory, while those of n-3 PUFAs are beneficial for neuronal functions. High levels of n-6 PUFAs are generally pro-inflammatory, while those of n-3 PUFAs are beneficial for neuronal functions [37, 38]. Deficiencies of n-3 PUFAs and an increased n-6/n-3 PUFA ratio in erythrocytes have been reported in humans with mental disorders including ASD [39–41]. The reduction in unsaturated fatty acid content that we observed in this work in both rat ASD models can therefore be an element of the pathomechanisms of behavioral changes and a candidate ASD biomarker.

In the present study, we noticed that in the THAL group, estriol and testosterone levels were decreased by 30% and 47%, respectively, whereas progesterone levels were increased. A slightly different pattern of changes was found in the VPA group, where estriol levels were reduced by 22%, while testosterone levels were increased by 65%. Estriol is produced from estradiol by the cytochrome P450 family 1 subfamily A polypeptide (data from KEGG - Kyoto Encyclopedia of Genes and Genomes). Estriol is the only form of estrogen we could identify as an isolated NMR signal, and therefore, it serves as an indicator of the content of estrogen in the brain. Estrogen is one of the main regulators of brain energy metabolism [42] and coordinates functional interactions among organs, cells, and genes [43]. Estrogen is synthesized in the ovaries and adrenal glands and in the brain, where it can also be synthesized from cholesterol [44, 45]. Regardless of gender, estrogen receptors are present in many parts of the brain, mostly in the hippocampus and cerebral cortex, both in neurons and in glial cells [46].

We hypothesize that in the THAL group, there are disorders of enzyme activity involved in converting progesterone to testosterone and estradiol, while the changes observed in the VPA group could be explained by the dysfunction of aromatase, an enzyme converting testosterone into estradiol. There have been reports of impaired functioning of this enzyme in patients with ASD [47]. Male and female sex hormones differentially regulate the expression of a novel autism candidate gene, retinoic acid–related orphan receptor-alpha (RORA), which transcriptionally regulates aromatase [48]. Hypothetically, sex steroids modulate the E/I balance, and changes in their concentration may sensitize the male brain to ASD-inducing factors [5]. Estrogens modulate GABA signaling by regulating the expression of glutamic acid decarboxylase [49] or the potassium-chloride cotransporter KCC2 [50], while androgens lead to GABA_A_-mediated excitotoxicity in the developing hippocampus of male rats [51].

Our study also showed elevated cholesterol levels and various changes in the content of its derivatives in both ASD models. Cholesterol is a precursor to steroid hormones, suggesting a hypothetical explanation that altered cholesterol metabolism can cause disturbances in sex hormone levels. The role of cholesterol metabolism disorders and the participation of their metabolites, such as testosterone, estrogen, cortisol, and vitamin D, in the pathogenesis of ASD have been suggested [52]. Cholesterol participates in numerous functions of the cell membrane, regulating, among others, permeability and fluidity [53]. The cellular level of free cholesterol is strictly regulated by a network of transcriptional and post-translational mechanisms sensitive to levels of free cholesterol and oxysterols (oxidized cholesterol derivatives), which can lower cholesterol through a negative feedback mechanism and prevent its toxic effects [54, 55]. 25-Hydroxycholesterol (25-HC), which was detected in our study, reduces free cholesterol by increasing cholesterol esterification by acyl-CoA-cholesterol acyltransferase in the endoplasmic reticulum [56]. We also detected 24(S)-hydroxycholesterol (24(S)-HC), which is the major brain cholesterol metabolite produced by cholesterol 24-hydroxylase. This pathway is crucial for brain cholesterol metabolism [57]. Unlike free cholesterol, 24(S)-HC is membrane permeable and thus could be metabolized in the periphery [58]. These dependencies were reflected in the decrease in the level of 24 (S) -HC in both experimental groups with an increase in the level of free cholesterol, which indicates a decrease in cholesterol metabolism via 24-hydroxylase cholesterol. Moreover, when considering the mechanisms of a hypothetical excitation/inhibition (E/I) imbalance in autism, modulation of NMDAR activity by oxysterols should be taken into account. 24(S)-HC is a selective and strong positive allosteric modulator of NMDARs, while 25-HC antagonizes this effect [59], providing neuroprotection also an NMDAR-independent mechanism [60]. A reduced level of 24(S)-HC, which we noticed in both experimental groups, is not consistent with the hypothesis being tested.

Many of the changes in the content of hydrophilic substances in the hippocampus shown in this work can also be referred to the hypothesis about the role of E/I imbalance in the pathogenesis of ASD, which suggests that, in autism, brain stimulatory glutaminergic neurotransmission prevails over inhibitory GABAergic neurotransmission [61–63]. This has been partly supported by the results of our previous studies using the same rat models of autism [30]. Abnormalities in the gene encoding the glycine receptor α2 subunit have been observed in a boy with autism [64]. In the current study, an increase in the content of glycine, taurine, and alanine in the hippocampus in the THAL model was noticed, while in the VPA model, it was slightly reduced, but the level of GABA increased (Table 2). GABA, as well as glycine, taurine, and alanine, are tonic agonists of GABA_A_ and glycine receptors, respectively, which are coupled to chloride channels and in the adult brain play the role of major inhibitory neurotransmitters [65, 66]. However, this is a more complex issue because, in the early stages of development, GABA and glycine depolarize neurons due to the relatively high intracellular concentration of Cl− ions, but during development, GABA and glycine function shifts from excitatory to inhibitory neurotransmitters [67–69]. It has been suggested that a delay in this shift may result in neurodevelopmental disorders, including ASD [70]. This may also apply to animal, pharmacological ASD models.

In this study, we observed a decrease in guanine content and an increase in GMP/IMP concentration in the hippocampus in the THAL model. These results may be related to the pathogenesis of autism because urine metabolomics studies of autistic children indicated impaired purine transformation [71], and may indirectly point to neurotransmission disorders and E/I imbalance in this ASD model. The guanine-based purinergic system (GBP) has many functions in nerve cells, including the modulation of NMDA receptor activity important in protecting against excitotoxicity. Moreover, GMP reduces glutamate binding to receptors (for review, see [72]), guanosine, a guanine substrate in the purine metabolic cycle (KEGG), prevents glutamate release in hippocampal slices [73], and GBP interacts with the activity of the glutamate transporter [74]. Chronic administration of GMP reduces the expression of NMDA and AMPA receptor subunits and the glutamate transporters EAAC1 and GLT-1 in the rat cerebral cortex [75].

The abovementioned disturbances in brain amino acid concentrations in both rat ASD models also concern the neuropeptide N-acetylaspartylglutamate (NAAG), an endogenous agonist of presynaptic metabotropic glutamate receptor 3 (mGluR3) which inhibits glutamate release. In the VPA model, the NAAG level and the levels of aspartate and NAA, which are NAAG precursors, were lower than those in the control group. In the THAL model, however, the NAA level was increased, while NAAG was decreased. These results could indicate a deficiency in the activity of N-acetylaspartylglutamate/N-acetylaspartylglutamylglutamate synthase or the hyperactivity of glutamate carboxypeptidase II (KEGG). Although there is no data in the literature linking disturbances in NAA/NAAG levels with ASD, they were observed in schizophrenia [76, 77].

### Hypotheses of Impaired Energy Metabolism and Oxidative Stress in the Brain in ASD

In our studies using proton and phosphorus NMR spectroscopy, we did not observe changes in the spectra of the ATP, ADP, and AMP signals due to the low concentrations of these substances, below the detection limit of the method. Also, the acetyl-CoA molecule is not detectable. However, increased lactate levels observed in the VPA group may be a consequence and an indicator of energy deficit. Lactate is an important intermediary in numerous metabolic processes [78, 79] and a preferred neuronal fuel [80, 81]. In the VPA model, reduced levels of methylmalonate and acetate and a deficiency in pyruvate, 2-oxo-3-hydroxyisovalerate, 2-oxoisovalerate, L-leucine, and L-valine were also noted, which can be hypothetically explained by acetyl-CoA deficiency (KEGG). Another circumstantial evidence indicating a possible energy deficit is the elevated levels of ketone bodies in the THAL model. Typically, ketone bodies are converted in the brain to acetyl-CoA, which can then enter the citric acid cycle to produce ATP (KEGG). The ketone body β-hydroxybutyrate has been shown to depolarize the plasma membrane and interfere with the synaptic vesicle cycle [82], which shows the possible functional consequences of this phenomenon.

An increase in the level of hypoxanthine in the hippocampus in both rat ASD models can lead to disorders in adenosine transport and imbalances in the activity of adenosine, dopamine, and serotonin receptors, as well as to abnormalities in the development of neurons, resulting from altered expression of some genes responsible for early neuronal differentiation [83, 84]. Moreover, this effect is indirectly indicative of energy deficit. Hypoxanthine has been shown to induce energy disorders in the brain [85]; its intrastriatal administration altered neuroenergetic parameters and caused mitochondrial dysfunction and apoptotic cell death [86]. These disorders and a decrease in ATP levels appear to be associated with oxidative stress because they may be prevented by pretreatment with free radical scavengers [87].

The results of several studies indicate a possible contribution of oxidative stress to the pathomechanisms of ASD [88–90]. A number of our results, such as the increase in hypoxanthine level in the THAL model discussed above, are consistent with this hypothesis. It is known that increased levels of hypoxanthine may lead to an increase in the production of reactive oxygen species and an exacerbation of the oxidative stress response [91, 92], leading to impairment of brain energy metabolism [93]. Oxidative stress is indicated by a decrease in the concentration of glutathione (GSH), which is a natural free radical scavenger in the cell, in the VPA model, and a similar trend in the THAL model. Moreover, in the THAL model, we found an increase in the level of 8-hydroxyadenine, which is a marker of DNA damage that may be a consequence of GSH deficiency [94, 95].

In our studies, hypoxanthine and allantoin which are markers of free radical production [96] were elevated in the THAL model.

### Other Hydrophilic Compounds and Their Possible Role in ASD Pathomechanisms

In both animal models, we observed changes in the levels of many other hydrophilic compounds that can affect neuronal function and contribute to the development of autism. In the THAL model, we noticed increased levels of choline (Cho) and creatine (Cr) and the Cho/Cr ratio, while in the VPA model, they were lowered. The results of clinical studies also differ; both a significant increase in the Cho/Cr ratio [97] and a decreased Cho/Cr levels in the brains of ASD children [98] were reported.

In the brains of rats from the THAL group, an increased level of myo-inositol and a reduced level of scyllo-inositol were found, which indicate disturbances in the metabolism of inositol, an important carbocyclic sugar that is involved in cellular signal transduction and osmoregulation. Preclinical studies showed positive effects of scyllo-inositol supplementation in in vivo and in vitro models of Alzheimer’s disease (AD) [99], while inositol supplementation in individuals with ASD yielded negative results [100]. We also noticed increased valine, leucine, and isoleucine levels in both ASD models, which may be due to the dysfunction of the enzyme amino acid transferase (KEGG). A reduction in the levels of 3-hydroxyisovalerate and methyl malonate confirms this interpretation. Similar disturbances in the levels of these amino acids in the urine of autistic children have been reported [101]. Reduced blood valine levels have been observed in children with propionic acidosis and autistic features [102].

## Conclusions

Although it is known that very similar behavioral symptoms that correspond to symptoms in autistic patients are observed in the two ASD rat models used in these studies, our NMR spectroscopic analysis showed large differences between these models in the content of several metabolites in the hippocampi. Table 4 provides a summary of the abovementioned compounds whose levels have been changed, directions of change, and metabolic pathways in which they are involved. We believe that these differences between VPA and THAL models could be a reflection of the spectrum of phenotypes observed in patients with ASD. Despite these differences, the following metabolic pathways were identified that were disrupted in both ASD models: steroid hormone biosynthesis; FAs biosynthesis; the synthesis and degradation of ketone bodies; glycerophospholipid metabolism; cholesterol metabolism; purine metabolism; arginine and proline metabolism; and valine, leucine, and isoleucine biosynthesis and degradation. These results may indicate disturbances in energy production, altered cell membrane structure, disturbances in excitatory and/or inhibitory neurotransmission, and the induction of oxidative stress in the hippocampus. The causal relationship between these disorders, the primary triggering mechanism(s), or their role in behavioral disorders similar to autism remains unclear. However, the obtained results may be useful in the future in the choice of the optimal animal model for ASD studies. In addition, these results may be advantageous for interpreting the results of our ongoing metabolomics studies using body fluids collected from rats from both ASD models.

**Table 4:** Functions and metabolic pathways of metabolites (VIP > 1) in two models of autism; directions of changes found in each of the models compared with the control are presented

Metabolites	Metabolic pathway	Probably pathomechanism	VPA model	THAL model
Progesterone	Steroid hormone biosynthesis	Energy and neurotransmission disturbances	↓	↑
Testosterone			↑	↓
Estriol			↓	↓
FA, MUFA, PUFA	FA biosynthesis	Energy production disturbances, cell membrane functional disturbances	↓	↓
Lysophosphatidylcholine	Glycerophospholipid metabolism	Cell membrane functional disturbances	↓	↑
Cholesterol	Cholesterol metabolism	Neurotransmission disturbances		↑
Cholesterol ester				↑
25-Hydroxycholesterol				↑
24-Hydroxycholesterol			↓	
8-Hydroxyadenine	Purine metabolism	Oxidative stress	↓	↑
Hypoxanthine			↑	↑
GMP/IMP			↓	↑
Allantoine			↓	↑
Guanine, xanthine				↓
Glycine	Glycine, serine metabolism		↓	
Threonine	Threonine metabolism	Neurotransmission disturbances		↑
Glutathione	Glutathione/cysteine and methionine metabolism	Oxidative stress	↓	
Phosphorylcholine	Glycerophospholipid metabolism	Neurotransmission disturbances		
				↑
Phosphoethanolamine				↑
Glycerophosphorylcholine				↑
Choline				↑
Creatine	Arginine and proline metabolism	Neurotransmission disturbances Oxidative stress		↑
Creatinine			↑	↑
GABA				↑
Taurine	Taurine, alanine and glutamate, pyruvate metabolism	Neurotransmission disturbances Oxidative stress		↑
Aspartate				↑
Cysteic acid				↑
Lactate				↑
NAA				↑
NAAG			↓	↓
Glycolic acid	Glyoxylate and dicarboxylate metabolism	Oxidative stress	↓	
Acetate	Synthesis and degradation of ketone bodies	Energy production disturbances		↑
3-Hydroxybutyrate			↓	
Myo-inositol	Inositol metabolism	Energy production disturbances		↑
Scyllo-inositol				↓
3-Hydroxyisovalerate	Valine, leucine, isoleucine biosynthesis Pyrimidine metabolism	Neurotransmission disturbances Oxidative stress		↓
Thymine				↑
Methylmalonate				↓
2-Oxoisovalerate	Valine, leucine, isoleucine degradation	Neurotransmission disturbances Oxidative stress		↑
Valine, leucine, isoleucine				↑
